# Gastric-filling ultrasonography to evaluate gastric motility in patients with Parkinson's disease

**DOI:** 10.3389/fneur.2024.1294260

**Published:** 2024-02-12

**Authors:** Xianwei Zou, Xiaqing Chen, Yanxia Wen, Xiaofeng Jing, Man Luo, Fengyue Xin, Yao Tang, Mengfei Hu, Jian Liu, Fan Xu

**Affiliations:** ^1^Department of Neurology, First Affiliated Hospital of Chengdu Medical College, Chengdu, Sichuan, China; ^2^Department of Public Health, Chengdu Medical College, Chengdu, Sichuan, China; ^3^Department of Ultrasonography, First Affiliated Hospital of Chengdu Medical College, Chengdu, Sichuan, China

**Keywords:** gastric emptying, gastric-filling ultrasonography, Parkinson's disease, cross-sectional area, gastric motility, symptom fluctuations

## Abstract

**Background:**

Delayed gastric emptying is a common non-motor symptom of Parkinson's disease (PD). However, there is currently no objective evaluation and diagnostic method for this condition.

**Objectives:**

The purpose of this study was to evaluate the feasibility of gastric-filling ultrasonography for gastric motility in patients with PD and the relationship between gastric dynamics and gastrointestinal symptoms and motor symptoms of PD.

**Design, setting, and patients:**

We performed a case-control study with 38 patients with PD and 34 healthy controls.

**Methods:**

All patients underwent a 120-min ultrasonography examination using a 500-ml semi-liquid test meal. We determined the antral contraction amplitude (ACA), the antrum contraction frequency (ACF), the motility index (MI), and the gastric antral cross-sectional area (CSA). We acquired the CSA at six time points: fasting for 12 h (T0), immediately after drinking the semi-liquid test meal (T1); and at 30 (T30), 60 (T60), 90 (T90), and 120 (T120) min. We calculated the gastric emptying rate (GER) at different time points by using the CSA. We compared the GER between the groups and evaluated the correlation between the GER and gastrointestinal symptoms and motor symptoms of PD.

**Results:**

The MI and ACF were significantly lower in the PD group compared with the control group (*P* < 0.05). The GER at T30 and the ACA showed no significant difference between the groups (*P* > 0.05). At different time points, the GER was significantly different between the PD and control groups (*P* < 0.001). There was no significant association between the GER and gastrointestinal symptoms; none of them were risk factors for impaired gastric emptying (odds ratio > 1). The GER was negatively correlated with the severity of PD motor symptoms (*P* < 0.05).

**Conclusion:**

Patients with PD had significantly delayed gastric emptying, which was negatively correlated with the severity of PD motor symptoms. Measuring gastric emptying by gastric-filling ultrasound had good diagnostic value in clinical screening for delayed gastric motility in patients with PD.

**Clinical Trial Registration:**

https://www.chictr.org.cn/showproj.html?proj=126304.

## 1 Introduction

Parkinson's disease (PD) is a chronic neurodegenerative disease with prominent motor and non-motor symptoms (1–3). About 70% of patients with PD have gastric dysmotility, a common non-motor symptom of PD, that manifests as bloating, nausea, vomiting, malnutrition, and weight loss (4–8). This conditions leads to a significant decline in the quality of life of patients. Meanwhile, delayed gastric emptying may contribute to fluctuations in drug absorption rates, and these fluctuations affect drug efficacy (9, 10). Most antiparkinsonian drugs, such as levodopa, are delivered orally (11–14). After administration, the drug is absorbed in the proximal small intestine, and its effective dosage depends on the gastric emptying rate (GER). Delayed gastric emptying may delay the peak in the blood concentration, which may contribute to the fluctuations in PD motor symptoms. Therefore, screening and evaluating gastric motility in patients with PD is essential, but it has not attracted the attention of clinicians.

Gastric motility examination is not included in routine screening; one reason is the lack of adequate evaluation methods. While gastric scintigraphy is widely available to detect gastric motility and is considered the gold standard for diagnosis (15–17), it exposes the patient to considerable radiation and has a high false positive with the liquid phase. Therefore, it is only used in scientific research. As a simple, non-invasive, non-radioactive, and reproducible inspection method, gastric-filling ultrasonography has significant clinical advantages (18, 19). At present, clinical studies are using gastric-filling ultrasonography to determine gastric motility in patients with functional dyspepsia (20, 21), esophageal achalasia (22), and diabetes (23); in pregnant women (24, 25); and in children (26). However, there are no clinical trials using ultrasonography to evaluate gastric motility in patients with PD. Therefore, we used gastric-filling ultrasonography to assess gastric motility in these patients and explored whether it is a reliable and effective screening tool. We also evaluated the correlation between the GER and PD motor symptoms and gastrointestinal symptoms to explore whether patients with PD motor symptoms or gastrointestinal symptoms should receive routine screening of gastric motility.

## 2 Methods

### 2.1 Participants

From October 2019 to June 2020, 38 patients diagnosed with idiopathic PD were recruited from the neurology department of the First Affiliated Hospital of Chengdu Medical College. Thirty-four age- and sex-matched healthy controls (HC) were recruited from Health Management Center. Patients with PD fulfilled the UK Parkinson's Disease Society Bank Criteria (27). The exclusion criteria (28–31) were: (i) parkinsonism-plus syndromes and secondary parkinsonism; (ii) diabetes mellitus and gastrointestinal surgery and hypothyroidism; (iii) taking medications that could affect gastric motility (e.g., non-steroidal anti-inflammatory drugs, aminophylline, and digitalis); (iv) reflux esophagitis, stomach or duodenal ulcer, chronic cholecystitis and pancreatitis, malignant tumors of the stomach, pancreas, or colon, and other invasive diseases of the stomach; and (v) long-term heavy drinking. The research protocol was approved by the China Registered Clinical Trials Ethics Review Committee (registration number: ChiCTR2100046360). All participants signed an informed consent form.

### 2.2 Clinical data collection

The patients with PD were evaluated during the “off” phase. Each patient was assessed using the Hoehr–Yahr scale (H&Y), the Unified Parkinson's Disease Rating Scale Motor Scale III (UPDRS), the Mini-Mental State Examination (MMSE), and the Non-Motor Symptom Questionnaire (NMSQ). For all participants, upper gastrointestinal symptoms were assessed at the screening visit with the Gastroparesis Symptom Index (GCSI) (32, 33), and lower gastrointestinal symptoms were assessed with the Wexner Constipation Scale (34) to quantify the severity of the gastrointestinal symptoms. A trained neurologist conducted the entire assessment.

### 2.3 Gastric-filling ultrasonography evaluation

All participants had fasted and not taken medications for at least 12 h. All patients with PD were in the “off” phase. A GE Voluson E8 ultrasound with a C1-5 transducer was used to examine the subjects (35). First, the participants consumed a 500-ml test meal of gastroenterultrasound developer comprising coix seed, yam, soybeans, tangerine peel, and rice within 4–5 min (50 g of gastroenterultrasound developer was boiled in water and then cooled to about 25°C). The probe used was a linear, low-frequency (2–5 MHz) curved array abdominal transducer, initially positioned parasagittally in the epigastric area with the subject lying in the right lateral position (36).

### 2.4 Gastric emptying index

**Figure 1:**
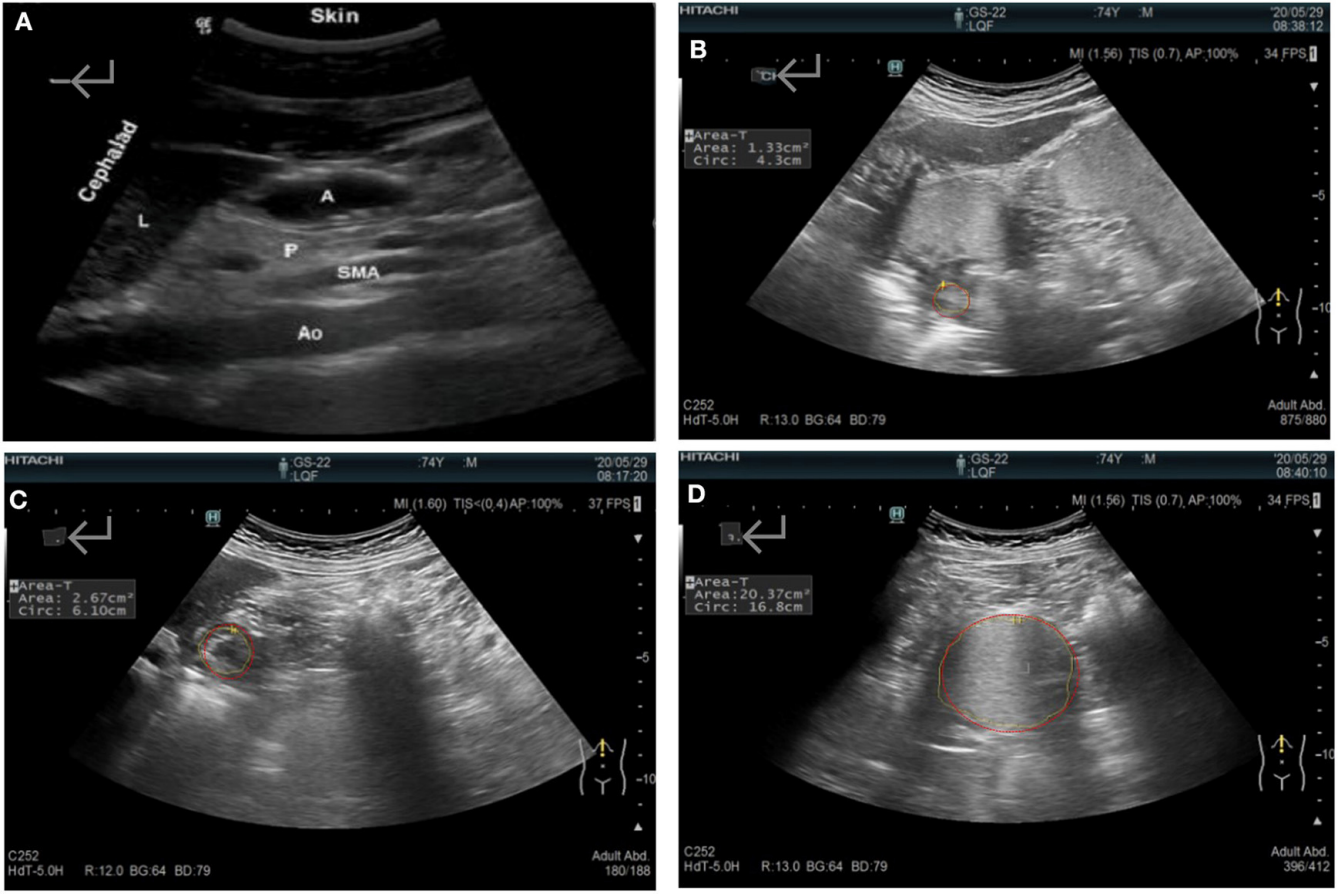
**(A)** Sonographic identification of the gastric antrum with the aid of anatomical landmarks: A, the gastric antral cross-sectional area; Ao, the abdominal aorta; L, the left lobe of the liver; P, the inferior pancreas; and SAM, the superior mesenteric artery. **(B)** The systolic antrum cross-sectional area. The gastric antrum cross-sectional area after **(C)** fasting and **(D)** immediately after a meal.

The gastric antral cross-sectional area (CSA) of the gastric sinus was used to evaluate the gastric emptying rate (GER). The antrum was located after identifying the superior left lobe of the liver, the major abdominal blood vessels (the abdominal aorta and the superior mesenteric artery or vein), and the inferior pancreas (Figure 1), as described previously. The area and diameters were measured in the serous layer at the following time points: after fasting for 12 h (T0); immediately after drinking the semi-liquid test meal (T1); and at 30 (T30), 60 (T60), 90 (T90), and 120 (T120) min after consuming the semi-liquid test meal. The GER was calculated as: [(CSA at T1 – CSA at T30/T60/T90/T120)/(CSA at T1 – CSA at T0)] × 100 (%). All of the scans performed were performed by the same experienced sonographer, as the force of ultrasound probe placement may have an effect on the interpretation of the cross-section (37).

### 2.5 Gastric motility index (MI)

Gastric motility was assessed by determining the contractile movement of the gastric antrum as an index, including the antral contraction amplitude (ACA), the antrum contraction frequency (ACF), and the gastric MI. We start timing at the end of the last gastric emptying and count how many gastric emptying cycles there are in 1 min is the ACF frequency. The ACA was calculated as (Sd – Sc)/Sd, where Sd is the maximum gastric ACA (in the middle of the antrum without contraction) and Sc is the minimum gastric ACA area (in the middle of the antrum when contracted). The ACF is the number of contractions of the gastric antrum per minute. Finally, the MI was calculated as ACA × ACF (38).

### 2.6 Statistical analysis

Demographics and baseline characteristics data were expressed as the mean (standard deviation) and N (%), the analysis between two groups by using two-tailed student's *t*-test and chi-square test. The GER was compared between the PD and HC groups with repeated measures analysis of variance. Logistic regression was used to analyse the relationship between the GER and dyspepsia symptoms, with dyspepsia symptoms as the dependent variable and the GER as the independent variable. The relationship between the GER and PD motor symptoms was determined with Spearman rank correlation analysis. Analyses were performed with Stata SE 15 and Graphad Prism 9.0. *P* < 0.05 was considered statistically significant.

## 3 Results

### 3.1 Comparison between the groups

From August 2019 to September 2020, we enrolled a total of 72 patients. Table 1 shows the demographics and baseline characteristics of the groups. Sex, smoking, drinking, and age not different between the PD and HC groups (*P* > 0.05). The ACA was not different between the groups (*P* > 0.05). The ACF and MI were significantly lower in the PD group compared with the HC group (*P* < 0.05). Therefore, the main influencing factor on the GER is the ACF, not the ACA. Subgroup analysis was prone to bias and random effects due to small numbers, so we did not stratify the patients based on disease severity.

**Table 1:** Demographics and baseline characteristics.

**Demographics**	**Parkinson's disease**	**Healthy control**	***P***
	**(*****n*** = **38)**	**(*****n*** = **34)**	
Female sex	22 (57.89)	21 (61.76)	0.74
Smoking	7 (18.42)	6 (17.65)	0.93
Drinking	7 (18.42)	6 (17.65)	0.93
Age (years)	70.26 ± 7.66	70.91 ± 7.40	0.72
ACA	13.56 ± 4.67	12.71 ± 3.18	0.375
ACF	2.53 ± 0.73	3.32 ± 0.53	<0.001
MI	35.12 ± 15.12	41.71 ± 9.76	0.033
Disease duration (years)	4.15 ± 2.87 (0.5–11.0)	NA	NA
UPDRS III	30.50 ± 15.46	NA	NA
Hoehn–Yahr	2.50 ± 0.82	NA	NA
GCSI	2.47 ± 4.89	NA	NA
Wexner	4.08 ± 5.65	NA	NA

The data are presented as the mean ± standard deviation or the number (per cent).

ACA, Antral contraction amplitude; ACF, Antrum contraction frequency; MI, motility index; UPDRS III, Unified Parkinson's Disease Rating Scale III; GCSI, Gastroparesis Symptom Index.

### 3.2 Comparison of the GER between the groups

**Figure 2:**
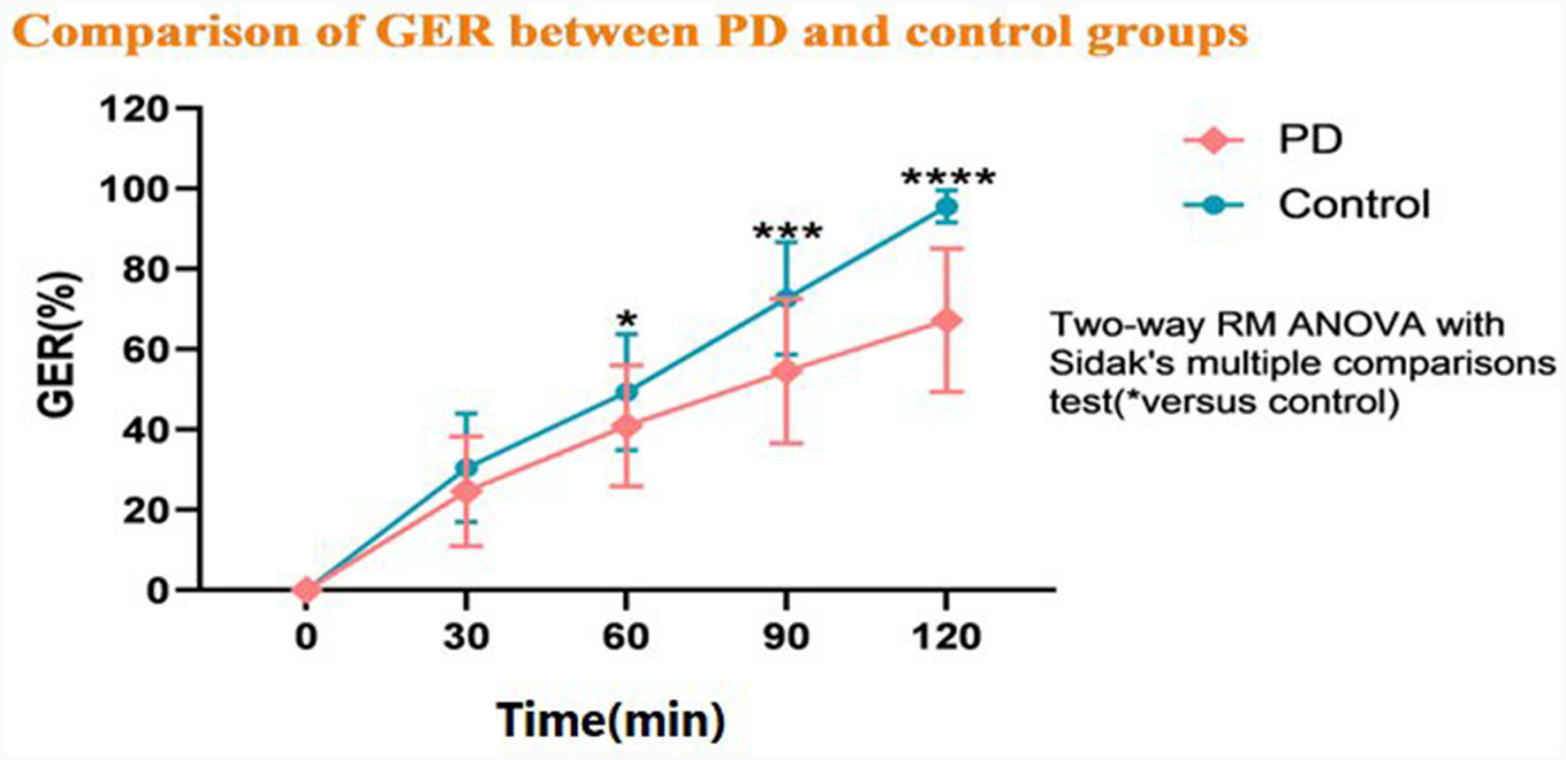
Comparison of the gastric emptying rate (GER) between the Parkinson's disease (PD) and healthy control groups. ^*^P < 0.05, ^**^P < 0.01, ^***^P < 0.001, and ^****^P < 0.0001.

The GER was slower in the PD group than in the HC group at T60, T90, and T120 (*P* < 0.05), but there was not a significant difference between the groups at T30 (*P* > 0.05). As shown in the gastric emptying curve (Figure 2), the GER presented a gradual upward trend in the HC group. The rate of growth was slower in the PD group compared with the HC group, and the growth between 90 and 120 min was significantly slower than the HC group. Besides, the MI was significantly different between the PD and HC groups at different time points (Table 2).

**Table 2:** Comparison of gastric motility index between the Parkinson's disease and healthy control groups.

**Source**	**SS**	**df**	**MS**	**F**	***P***
Model	168,844.27	74	2,281.6793	18.73	<0.001
Individual	55,236.836	71	777.98361	6.39	<0.001
GER stage	113,607.43	3	37,869.144	310.82	<0.001
Residual	25,951.216	213	121.83669		
Total	194,795.48	287	678.72991		

df, degrees of freedom; GER, gastric emptying rate; MS, mean squares; SS, sum of squares.

### 3.3 Relationship between gastrointestinal symptoms and the GER

There was no significant association between the gastrointestinal symptoms and the GER (odds ratio > 1, Table 3). Hence, none of them were risk factors for delayed gastric emptying.

**Table 3:** Logistic regression analysis of dyspepsia and GER.

**Influential factor**	***B***	**SE**	***Wald***	**OR**	**95% CI**	***P***
Nausea	−0.145	0.754	0.037	0.865	0.197–3.795	0.848
Retching	0.116	0.352	0.108	1.123	0.563–2.240	0.742
Vomiting	1.707	1.414	1.457	5.514	0.345–88.167	0.227
Stomach fullness	0.571	0.600	0.906	1.770	0.546–5.736	0.341
Not able to finish normal-sized meal	−0.511	1.135	0.203	0.600	0.065–5.541	0.652
Feeling excessively full after meals	−0.131	0.590	0.049	0.878	0.276–2.791	0.825
Loss of appetite	0.411	0.748	0.301	1.508	0.348–6.534	0.583
Bloating	−2.292	1.383	2.746	0.101	0.007–1.520	0.097
Stomach or belly visibly larger	−0.633	1.043	0.368	0.531	0.069–4.099	0.544
Constipation	−0.042	0.082	0.270	0.959	0.817–1.125	0.603

### 3.4 Relationship between PD motor symptoms and the GER

**Figure 3:**
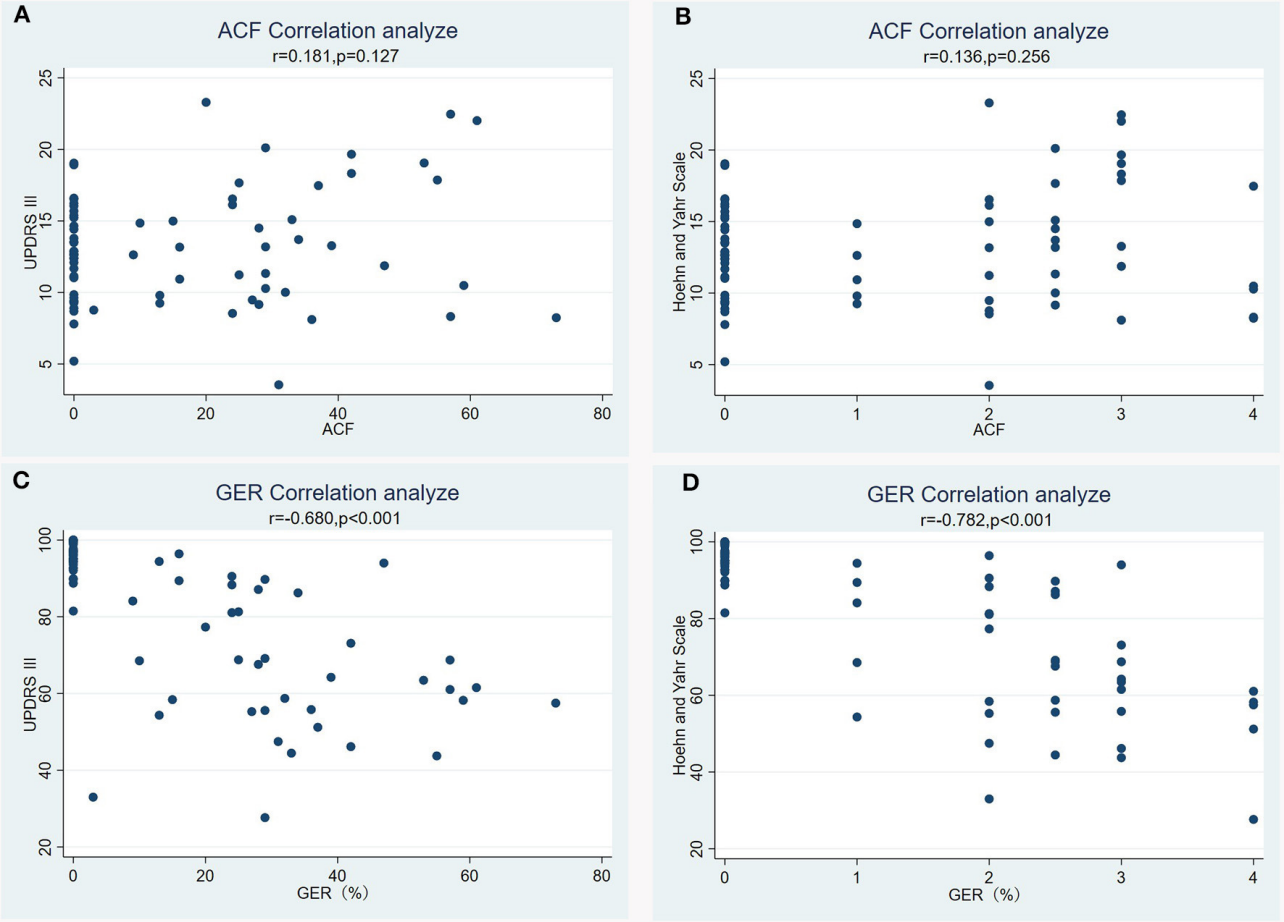
**(A–D)** Linear regression analysis of the relationship between Parkinson's disease motor symptoms and the gastric emptying rate (GER).

The GER was negatively correlated with the PD motor symptoms: the slower the GER, the more severe the PD motor symptoms (Figure 3). The severity of PD motor symptoms were assessed with the UPDRS III and the H&Y scale, with higher scores indicating more severe disease. At the same time, we also found no correlation between PD duration and the GER.

## 4 Discussion

Our results confirmed that patients with PD had significantly delayed gastric emptying, consistent with previous research (39, 40). Our findings also demonstrated that the HC group had more vigorous motor activity in the distal antrum, denoted by a higher ACF. This means that the main factor affecting the GER is the ACF, not the ACA. However, the reason is not apparent and requires further investigation. Another significant finding was the lack of a significant association between gastrointestinal symptoms and the GER, which was consistent with previous research (21). It has been found that in some patients with low UPDRS-III scores or early H&Y stage do not present with symptoms of gastric dysfunction, such as bloating, nausea, acid reflux, and decreased appetite (39), but ultrasonography examination reveals that the patient has already experienced a delay in gastric emptying. Objective gastric motility abnormalities are frequently detected even in PD patients without subjective gastrointestinal (GI) complaints. Therefore, early screening and timely intervention may delay the progression of the disease and reduce the development of motility complications. Objective gastric motility abnormalities are frequently detected even in patients in PD without subjective gastrointestinal complaints. Therefore, accurate screening methods are essential. In addition, we found that the GER was negatively correlated with PD motor symptoms. Previous studies have only associated the GER with the pharmacokinetics of levodopa in patients with PD without specifically exploring the association between the GER and PD motor symptoms. The association between the GER and PD motor symptoms may be as follows: first, delayed gastric emptying in patients with PD may delay and lower the peak plasma concentration of drugs (i.e., levodopa), thus aggravating motor symptoms. Second, with the aggravation of PD, there is increased gastrointestinal vagus nerve damage, leading to impaired gastric motility.

We used gastric-filling ultrasonography to investigate the effect of gastric motility. Previous reports have suggested that the gold standard method to evaluate gastric emptying is scintigraphy, but it has the disadvantage of radiation exposure. Wireless motility capsule smart pill (41) avoids radiation exposure but it has been less validated than scintigraphy and cannot be used in patients with a pacemaker or defibrillator. The carbon breath test using ^13^C-labeled octanoic acid (42, 43) is simple to perform, but it has poor accuracy: small intestinal bacterial overgrowth syndrome and aliphatic acid metabolism disorders in the liver can affect the experimental results. Studies have shown that many patients with PD have intestinal bacterial overgrowth syndrome (44–46), so a carbon breath test is not suitable to examine gastric motility in PD.

Recently, technological advances in magnetic resonance (MRI) have provided new tools for a non-invasive assessment of gastric function, such as a novel MRI-based three-dimensional model of stomach offers a detailed tool for evaluating gastric volumes, surface geometry, and wall tension (47). Using a four-dimensional (4D, volumetric cine imaging), free-breathing MRI sequence estimated multiple parameters describing gastric emptying, motility, and peristalsis propagation patterns (48). Another research shows the effectiveness of the proposed method for visualization and quantification of motility patterns from free-breathing dynamic MRI data (49). MRI may be more accurate, but they are expensive, complex, more time-consuming, and still have methodological limitations, making them inappropriate for routine clinical screening for impaired gastric dynamics in Parkinson's disease, as well as for patients who require dynamic monitoring of gastric emptying, such as those taking pro-gastric motivational medications, or observing motor complications (fluctuations and dyskinesias) in Parkinson's disease.

In 1980, Holt et al. (50) were the first researchers to use ultrasound to study normal people's total gastric contraction after a liquid test meal. Since then, ultrasound detection of gastric motility has grown continuously. At present, ultrasound has been used in many fields to screen patients with gastric motility disorders, and its accuracy has also been verified. Moreover, we have shown that gastric-filling ultrasonography is feasible to determine gastrointestinal dynamics in patients with PD.

Gastric dysfunctions are non-motor symptoms of PD that occur prior to the appearance of motor symptoms and persist at all stages of the disease (51, 52). It is unclear whether gastric dysfunction in PD is related to the primary central or peripheral neurodegenerative process. Myenteric neurons of the entire gastrointestinal tract represent one of the earliest sites of α-synuclein aggregation, the typical hallmark of PD, suggesting that alterations in the enteric nervous system of both upper and lower digestive tract could play a significant role in gastrointestinal motor alterations that occur in PD (53–55). Besides, delayed gastric emptying is associated with an impairment of the vagal brain–gut pathway (56), which involves the efferent fibers of the vagal pathway projecting directly to the gastric myenteric plexus. Deposition of α-synuclein in the enteric nervous system may lead to gastric vagal nerve damage and thus a series of neurotransmitter changes that delay gastric emptying (57–59).

Delayed gastric emptying may appear in patients with early-stage PD and can worsen as the disease progresses (60). Gastric motility can be dynamically monitored in patients with PD with ultrasonography to understand the gastric emptying status and to identify the early gastric dysmotility of this condition. This information could provide a useful and accurate objective basis for the treatment of PD with delayed gastric emptying. Simultaneously, if the patient has fluctuations in movement symptoms, especially the delayed opening period, then it should be considered to be related to delayed gastric empty, and gastrokinetic drugs should be added or the drug delivery route should be changed. This approach may be useful to ameliorate motor symptoms and their fluctuation in patients with PD.

Our study has several limitations. First, this trial was a case-control study. It was impossible to prospectively observe the effect of changes in gastric MI of patients with PD. Second, ultrasound is operator dependent and has a learning curve. Hence, all examinations need to be performed by the same person. Besides, patients cannot accept periodic examinations of up to 2 h in length. In the future, we will explore the relationship between delayed gastric emptying and motor symptoms in patients with PD through prospective studies.

In summary, gastric emptying is delayed to some extent in patients with PD. The gastric dysfunctions may aggravate the motor symptoms of the disease. Gastric-filling ultrasonography is simple, reproducible, non-radioactive, and accurate, and can distinguish the early gastric motility disorder of PD. Therefore, gastric motility in patients with PD, especially those with motor complications, could be routinely examined by using gastric-filling ultrasonography.

## Data availability statement

The raw data supporting the conclusions of this article will be made available by the authors, without undue reservation.

## Ethics statement

The studies involving humans were approved by China Registered Clinical Trials Ethics Review Committee. The studies were conducted in accordance with the local legislation and institutional requirements. Written informed consent for participation was not required from the participants or the participants' legal guardians/next of kin in accordance with the national legislation and institutional requirements.

## Author contributions

XZ: Data curation, Investigation, Methodology, Supervision, Writing – review & editing. XC: Data curation, Writing – original draft. YW: Data curation, Formal analysis, Writing – original draft. XJ: Data curation, Formal analysis, Writing – original draft. ML: Data curation, Investigation, Methodology, Writing – original draft. FXi: Data curation, Investigation, Methodology, Writing – original draft. YT: Data curation, Investigation, Methodology, Writing – original draft. MH: Data curation, Investigation, Methodology, Writing – original draft. JL: Data curation, Investigation, Methodology, Writing – original draft. FXu: Methodology, Supervision, Writing – review & editing.
